# Constitutive Activation of the Thyroid-Stimulating Hormone Receptor (TSHR) by Mutating Ile^691^ in the Cytoplasmic Tail Segment

**DOI:** 10.1371/journal.pone.0016335

**Published:** 2011-01-21

**Authors:** Zheng Liu, Feiyue Fan, Xiangjun Xiao, Yuanming Sun

**Affiliations:** 1 Tianjin Key Laboratory of Molecular Nuclear Medicine, Institute of Radiation Medicine, Chinese Academy of Medical Sciences and Peking Union Medical College, Tian Jin, China; 2 School of Biology, Georgia Institute of Technology, Atlanta, Georgia, United States of America; 3 Department of Epidemiology, MD Anderson Cancer Center, The University of Texas, Houston, Texas, United States of America; Alcon Research, Ltd., United States

## Abstract

**Background:**

Autosomal dominant non-autoimmune hyperthyroidism (ADNAH) is a rare genetic disorder of the endocrine system. Molecular genetic studies in ADNAH have revealed heterozygous germline mutations in the TSHR. To data, mutations leading to an increase in the constitutive activation of the TSHR have been described in the transmembrane segments, exoloops and cytoplasmic loop of TSHR. These mutations result in constitutive activation of the G_αs_/cAMP or G_αq/11_/inositol phosphate (IP) pathways, which stimulate thyroid hormone production and thyroid proliferation.

**Methodology/Principal Findings:**

In a previous study, we reported a new TSHR mutation located in the C-terminal domain of TSHR, which results in a substitution of the conserved Ile^691^ for Phe. In this study, to address the question of whether the I691F mutated receptor could be responsible for G_αs_/cAMP or G_αq/11_/IP constitutive activity, wild-type and TSHR mutants were expressed in COS-7 cells to determine cAMP constitutive activity and IP formation. Compared to the cell surface with expression of the A623V mutated receptor as positive control, the I691F mutated receptor showed a slight increase of cAMP accumulation. Furthermore, I691F resulted in constitutive activation of the G_αq/11_/IP signaling pathway.

**Conclusions/Significance:**

Our results indicate that Ile^691^ not only contributes to keeping TSHR inactive in the G_αs_/cAMP pathways but also in the G_αq/11_/IP cascade.

## Introduction

TSHR is a member of the superfamily of G-protein–coupled transmembrane receptors mediating most of their intracellular actions through G proteins [1]. The G protein family consists of the G_α_ and the tightly associated G_βγ_ subunits. There are four classes of G_α_ subunits: G_αs_, G_αi_, G_αq/11_ and G_α12/13_. Despite interaction of human TSHR with all four G_α_ subunits, biological relevance has only been attributed to the activation of G_αs_/cAMP, which is thought to regulate growth and differentiation of the thyroid; and to a lesser degree, to the activation of G_αq/11_/IP, which is thought to stimulate thyroid hormone synthesis and iodination [2], [3]. Familial non-autoimmune hyperthyroidism is an autosomal dominant genetic disease resulting from mutations in the TSHR. To data, mutations leading to an increase in the constitutive activation of the TSHR have been described in the first, second, third, fourth, fifth, sixth and seventh transmembrane segments, in the first, second, third exoloops and in the second and third cytoplasmic loop [4], [5], [6], [7], [8], [9], [10], [11], [12], [13], [14]. Some studies provided evidences that the third intracellular loop plays a critical role in G_αs_ protein activation [15]. In particular, mutation of the conserved Ala^623^ in third intracellular loop was reported to result in loss of TSH-stimulated IP formation leaving cAMP accumulation unaltered [16]. It suggests that the Ala^623^ could be important for specific G_αs_ protein coupling. Although it has been shown that receptor/G_αs_ protein interaction is determined by specific amino acids, knowledge about the key players in this selective interplay is still very limited.

**Figure 1:**
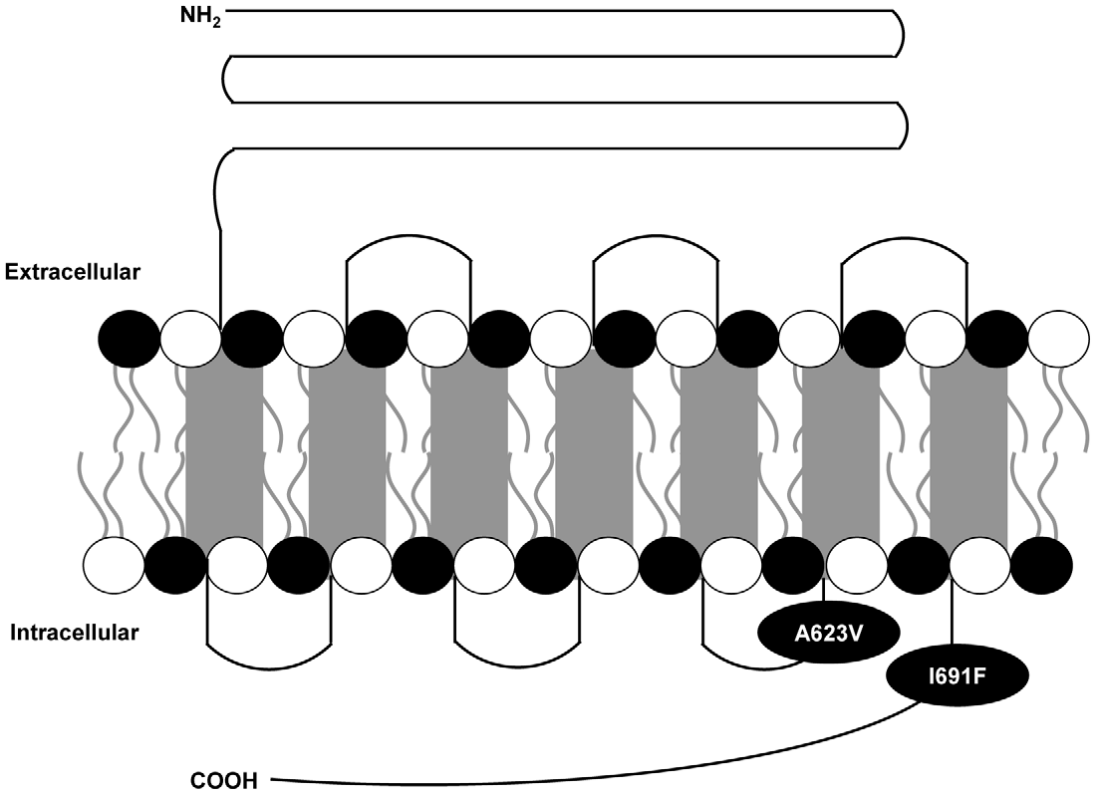
Schematic representation of the TSHR mutants (A623V and I691F). The mutants with A623V and I691F desciribed in this article. A623V and I691F locte in third intracellular loop and cytoplasmic tail region of TSHR respectively.

In a previous study, we collected a Chinese family with ADNAH across four generations. By mutation scan of the TSHR gene located within the region of interest, a heterozygous substitution (A→T) at position 2071 of the TSHR was found, changing isoleucine 691 to phenylalanine (Figure 1) [17]. In this study, using A623V as a positive control, we utilized a combination of functional assays, site-directed mutagenesis and image techniques to demonstrate that this mutation causes constitutive activation of G_αs_/cAMP and G_αq/11_/IP cascade signaling, which suggests the key role of isoleucine at position 691 in maintaining the inactive state between TSHR and G_α_ protein.

## Results

### Characterization of fluorescent proteins of wild-type and TSHR mutants

**Figure 2:**
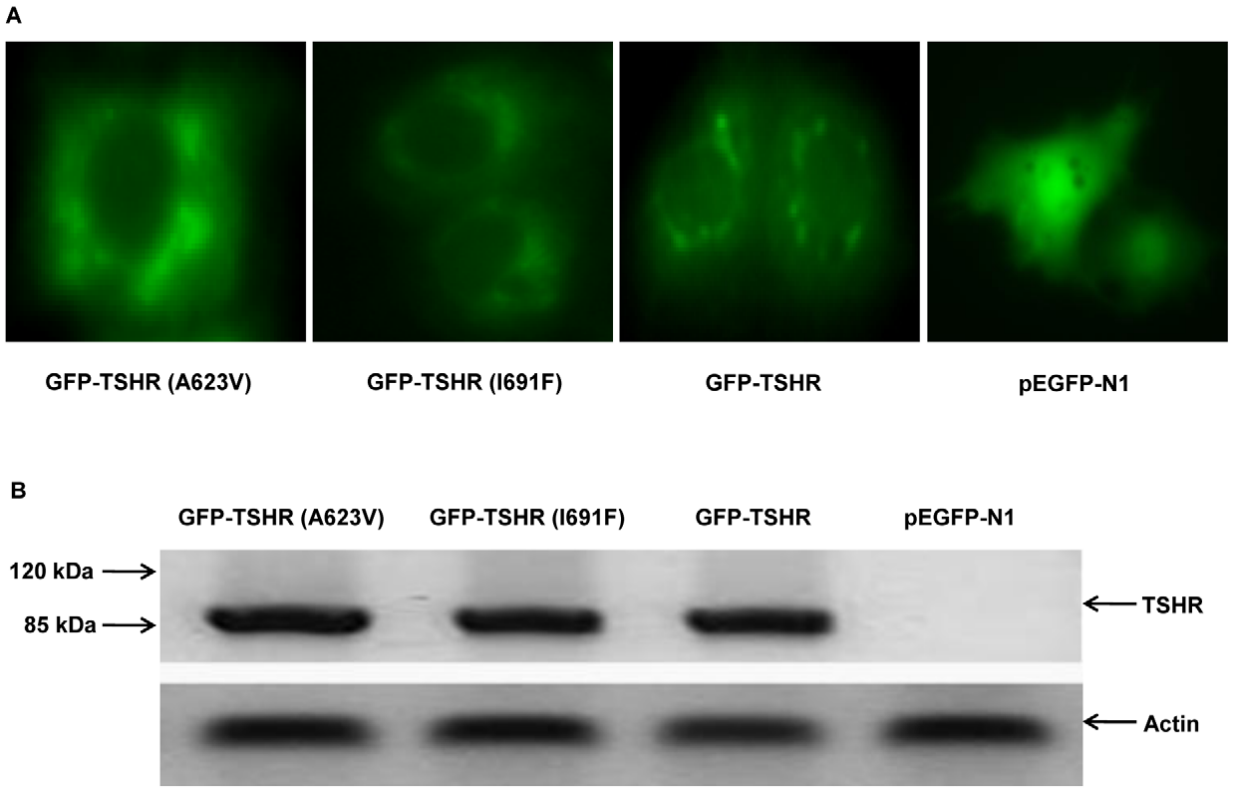
Characterization of chimeric constructs of TSHR and fluorescent reporters. (A) COS-7 cells transfected with GFP chimeras of wild-type or TSHR mutants and visualized by fluorescence microscopy. Wild-type TSH receptor is expressed at the plasma membrane. Mutant A623V and I691F receptors are poorly expressed at the plasma membrane and are retained in intracellular regions; and (B) Western blot analysis of different GFP chimeras using an anti-human TSHR antibody (H-115) antibody. The band consistent with the molecular weights of receptor (∼90 kDa, TSHR + GFP) forms were present.

By fusing GFP fluorescent reporters to the TSHR, we generated fluorescent chimeras of GFP-TSHR, GFP-TSHR (I691F) and GFP-TSHR (A623V) mutants to investigate their subcellular localizations by fluorescence microscopy. Wild-type TSHR chimeras were clearly expressed at the cell membrane. In contrast, I691F and A623V mutants' chimeras were retained in cytoplasm and poorly expressed on the plasma membrane (Figure 2A). To exclude the possibility that the intracellular retention of mutant receptors could be simply due to higher expression levels, a Western blot analysis was performed. The result shows that the total level of protein expression is similar for all constructs (Figure 2B).

### Cell surface expression

**Figure 3:**
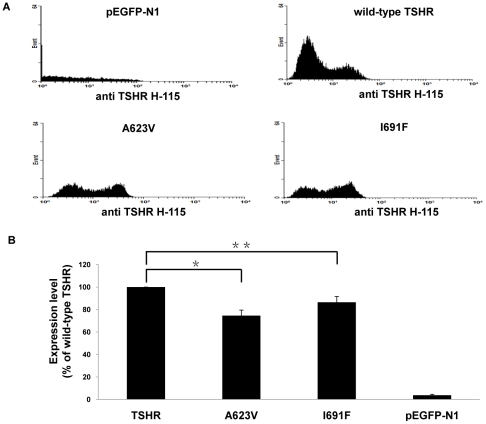
Cell surface expression determined by Flow cytometry analysis. (A) COS-7 cells were assayed after transfection with pEGFP-N1-vector, wild-type TSHR, mutants A623V and I691F. Fluorescence intensity is expressed in arbitrary units as a function of cell number plotted on a logarithmic scale. Representative mutants are shown. (B) Expression level of the pEGFP-N1, wild-type TSHR and mutants with A623V and I691F. Data are expressed as percentage expression of wild-type TSHR (set at 100%) and are presented as means ± S.E.M. of three independent experiments, each carried out in duplicate. ^*^ *p<*0.05; ^**^ *p<*0.001.

The level of receptor expression on the cell surface for the different constructs was independently measured by flow cytometry. Fluorescence intensity is expressed in arbitrary units as a function of cell number plotted on a logarithmic scale (Figure 3A). These two mutations led to a marked decrease expression of the mutated receptors on the cell surface compared to the wild-type TSHR (set at 100%), 74.6% for A623V and 86.4% for I691F respectively (Table 1, Figure 3B). The pEGFP-N1vector is not present on the cell membrane.

**Table 1:** Functional characterization of TSHR mutants.

Transfected constructs	Cell surface Expression (% of wild-type TSHR)	cAMP accumulation (Fold over wild-type TSHR basal)			IP accumulation (Fold over wild-type TSHR basal)		
		0 mU/ml bTSHBasal	10 mU/mlbTSH	100 mU/mlbTSH	0 mU/ml bTSHBasal	10 mU/mlbTSH	100 mU/mlbTSH
wild-type TSHR	100	1	3.2±1.12	8.2±1.27	1	2.2±0.7	6.1±0.12
A623V	74.6±4.9	5.8±0.3	9.6±1.2	16.3±1.4	0.3±0.04	0.7±0.02	0.9±0.1
I691F	86.4±5.2	3.9±0.08	6.4±0.19	12.1±1.1	2.7±0.2	7.3±1.1	8.2±1.9
pEGFP-N1	3.8±0.7	0.3±0.04	02.±0.02	0.4±0.06	0.2±0.01	0.3±0.03	0.3±0.01

### Basal and TSH-stimulated cAMP production

**Figure 4:**
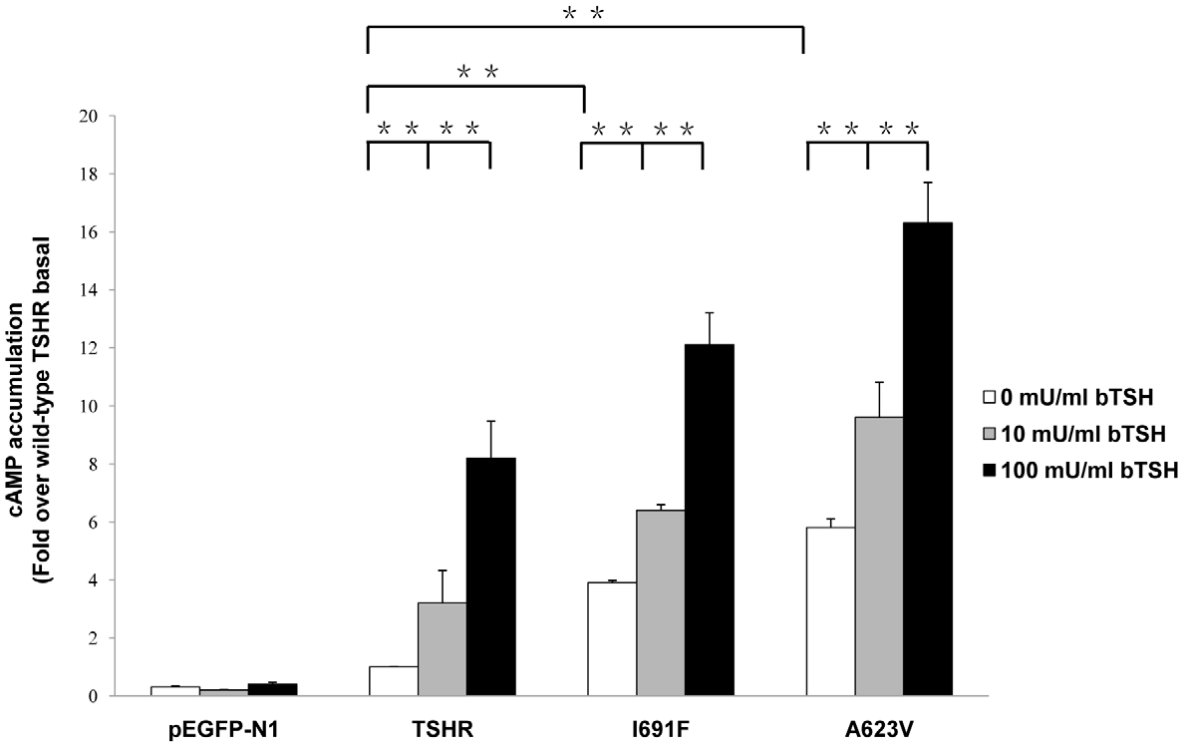
Basal and TSH-stimulated cAMP accumulation. cAMP accumulation assays were performed with transiently transfected COS-7 cells with pEGFP-N1-vector, wild-type TSHR and mutants with A623V and I691F. Forty-eight hours after transfection, COS-7 cells were incubated in 10 or 100 mU/ml bTSH. cAMP levels were determined as described in materials and methods. Data are expressed as relative to wild-type basal and given as means ± S.E.M. of three independent experiments, each carried out in triplicate. ^*^ *p<*0.05; ^**^ *p<*0.001.

Cells transfected with the I691F exhibited a 4 fold increase in basal cAMP accumulation with respect to cells transfected with wild-type TSHR, showing a constitutive activation (Table 1, Figure 4). Cells transfected with the mutants A623V displayed higher level (6 fold) of basal cAMP compared to the wild-type TSHR (set at 1). Furthermore, the biological response to bTSH of the cells transfected with the DNA of the mutated constructs was explored in terms of cAMP accumulation. I691F was able to produce a significant cAMP stimulation of 12 fold after 100 mU/ml of bTSH stimulation, which was lower than 16 fold that produced by A623V compared with the wild-type TSHR (set at 1) (Table 1, Figure 4).

### Basal and TSH-stimulated inositol phosphate formation

**Figure 5:**
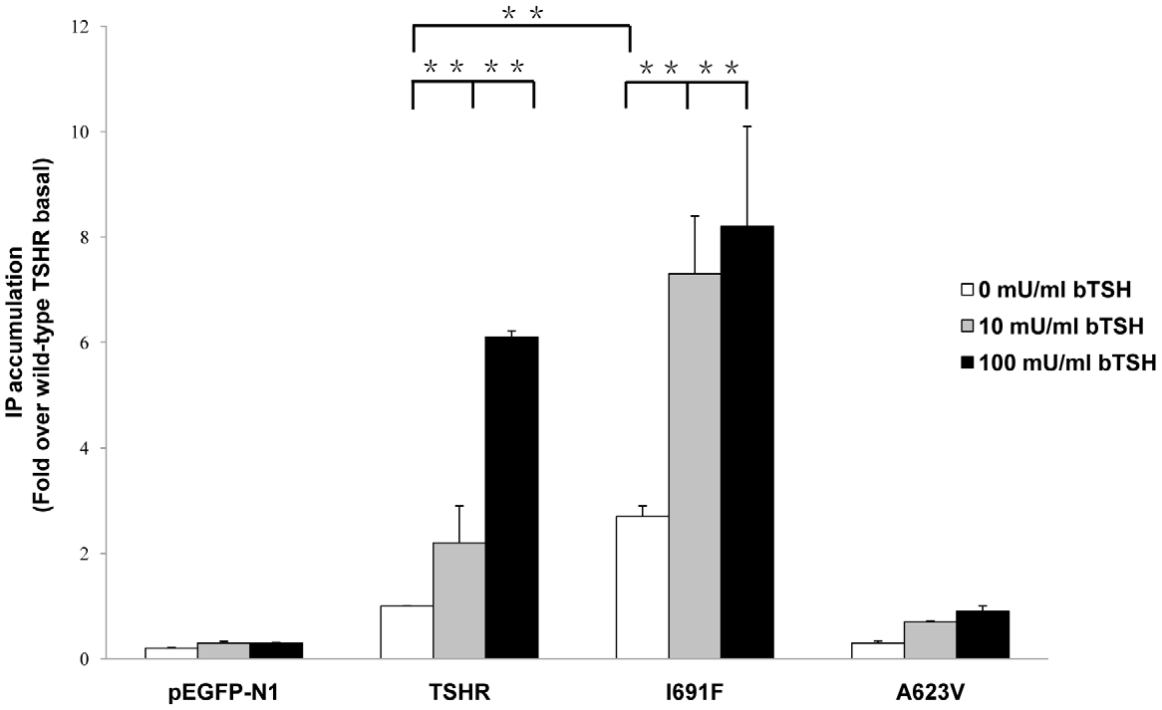
Basal and TSH-stimulated IP accumulation. COS-7 cells were transfeted with pEGFP-N1-vector, wild-type TSHR and mutants with A623V and I691F. Twenty-fourt hours after transfection, cells were incubated in 10 or 100 mU/ml bTSH. IP accumulation was determined as described in materials and methods. The basal IP production of the wild-type TSHR was set at 1 and all other data are expressed as fold of the basal IP production of the wild-type TSHR. Data are presented as means ± S.E.M. of two independent experiments, each carried out in triplicate. ^*^ *p<*0.05; ^**^ *p<*0.001.

As expected from previous studies, no significant increase of basal levels of IP production was observed in the cells transfected with A623V [16]. However, an increase was observed in cells transfected with the wild-type TSHR and I691F, showing a constitutive activity for the IP production. Cells transfected with the I691F mutant showed a slight increase of 3 fold in basal IP production with respect to wild-type TSHR (set at 1) (Table 1, Figure 5). Furthermore, stimulation of IP accumulation by 100 mU/ml of bTSH, I691F showed a significant increased production (8 fold) of IP with respect to the wild-type TSHR (set at 1) (Table 1, Figure 5).

## Discussion

Our finding supports the idea that the cytoplasmic tail region is a potential interaction site between TSHR and G_αs_ protein. The difference in basal cAMP level reflects the ability of the mutant receptors to activate the G_αs_/cAMP pathway [18]. The A623V construct had 1–2 fold higher levels in cAMP response than I691F. So, A623V mutation exhibited stronger constitutive activity, indicating that A623V critically required for productive G_αs_ coupling. Moreover, these results support the hypothesis that there are several active conformations of the TSHR, which differ in their capability to activate different downstream signaling cascades, as previously reported [19], [20], [21]. The vast majority of TSHR mutations activate the G_αs_/cAMP system, but only a few TSH receptor mutations can also activate the G_αq/11_/IP pathway, although the effects of this pathway in the pathogenesis of ADNAH remain unknown [22], [23]. In previous studies, the substitution of Ala^623^ with different amino acids lead to constitutive activation of the G_αs_/cAMP cascade and to a selective loss of G_αq/11_/IP signaling of the TSHR [16]. Our research also showed that A623V had full cAMP responses, but blunted IP responses. Interestingly, our work demonstrated I691F could active both G_αs_/cAMP and G_αq/11_/IP signaling pathways. These findings suggest that (1) Ile^691^ belongs to the cytoplasmic tail proposed to play a role in G_αq/11_/IP signal transduction; and (2) Ile^691^ is responsible for the maintenance of TSHR in the inactive state.

**Figure 6:**
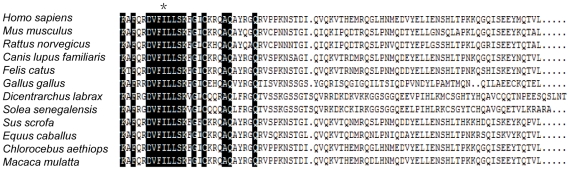
The alignment of amino acids in the cytoplasmic tail segment. Alignment of the cytoplasmic tail segment of the TSHR with different species, *Homo sapiens, Mus musculus, Rattus norvegicus, Canis lupus familiaris, Felis catus, Gallus gallus, Dicentrarchus labrax, Solea senegalensis, Sus scrofa, Equus caballus, Chlorocebus aethiops, Macaca mulatta*. The sequence of the cytoplasmic tail region of the TSHR between residues 688 and 695 including Ile^691^ (*) is highly conserved in various species.

Chazenbalk *et al*. showed that residues 709–764, two-thirds of the C-terminal domain of the cytoplasmic tail, can be removed without functional impairment of the TSHR suggesting that the cytoplasmic tail may play a nonessential role [24], [25]. However, other reports showed the half of the cytoplasmic tail (up to residue 721) is essential for full expression of functional activities [26]. Sequence comparison revealed that the C-terminal amino acids within 698–695 are highly conserved within the family of TSHR (Figure 6). In general, the substitution of isoleucine for phenylalanine would be considered as a ‘safe’ residue substitution in a protein because the change from isoleucine to phenylalanine represents a conservative substitution as both are nonpolar, hydrophobic amino acids [27]. However, phenylalanine is considerably bulkier as it has a benzene ring. This maybe disrupts the tight association of the amino acids and impairs folding and trafficking of the mutated receptors leading to constitutive activity of signaling pathways. Moreover, this molecular change may provide in the transformed cells a very strong signal, resulting not only in the growth advantage by stimulation of G_αs_/cAMP pathways, but also activation by G_αq/11_/IP pathways. This may eventually explain why the ‘safe’ mutation I691F causes ADNAH.

In conclusion, we identified a new mutation with the ability to constitutively activate both G_αs_/cAMP and G_αq/11_/IP pathways in the cytoplasmic tail region of the human TSHR. Although the role of cytoplasmic tail region of TSHR is not known very well, our data support the concepts that the cytoplasmic tail play a role in signal transduction and that Ile^691^ contributes in keeping TSHR inactive in both the G_αs_/cAMP and the G_αq/11_/IP pathways. Our finding could give a new insight into the molecular mechanisms of TSHR activation.

## Materials and Methods

### Generation of TSHR mutants

cDNA encoding the human TSHR was obtained by RT-PCR as described by Alberti [5]. In brief, genomic DNA was extracted from peripheral blood by DNA Extraction Kit (TaKaRa) according to the manufacturer's instructions. The RT-PCR was performed using the following primers, forward: 5′-GAG GAT GGA GAA ATA GCC CCG AG-3′ and reverse: 5′-GTG TCA TGG GAT TGG AAT-3′ [14], and then cloned into the eukaryotic expression vector pEGFP-N1 (Clontech) to generate the pEGFP-N1-TSHR. Mutation I691F and A623V were created by site-directed mutagenesis using QuikChange Site-Directed Mutagenesis kit (Stratagene) according to the manufacturer's instructions using expression plasmid pEGFP-N1-TSHR as a template. Vectors containing two mutants, A623V and I691F were generated by primers 5′- GAT ACC AAA ATT GTC AAG AGG ATG GCT- 3′, 5′- AGC CAT CCT CTT GAC AAT TTT GGT ATC - 3′and 5′- AGG GAT GTG TTC TTC CTA CTC AGC AAG - 3′, 5′- CTT GCT GAG TAG GAA GAA CAC ATC CCT - 3′ respectively. The PCR products were sequenced by ABI 3100 automatic sequencer.

### Cell culture and transient expression of TSHR mutants

COS-7 cells were maintained in Dulbecco's Modified Eagle Medium containing 10% fetal bovine serum and 100 µg/ml penicillin-streptomycin (Invitrogen) in a humidified atmosphere of 5% CO_2_ in air at 37°C. COS-7 cells were seeded into wells of 6-well dishes overnight and transfected with 250 ng of plasmid using Lipofectamine 2000 (Invitrogen), according to manufacturer's instructions. One day before transfection, 2–5×10^5^ cells were plated in 2 ml of growth medium without antibiotics, so that cells will be 80–90% confluent at the time of transfection. Cells transfected with the pEGFP-N1 vector were used as controls.

### Total protein extraction and Western blot analysis

The COS-7 cells were harvested 48 hours after transfection and then lysed for 1 hour on ice with lysis buffer to extract protein. In brief, 20 µg protein amounts were run on 10% SDS-polyacrylamide gels. Rainbow prestained molecular mass markers (Invitrogen) were used as the standard. After electrophoresis, the separated proteins were transferred to a 0.45 µm nitrocellulose membrane. The membranes were blocked with 5% fat-free milk in PBS buffer for 1 hour at room temperature. After being washed three times with PBS containing 0.1% Triton X-100, the membranes were incubated with mouse anti-human TSHR antibody (H-115) 1∶2000 or anti-Actin (C-2) 1∶3000 (Santa Cruz Biotech) in PBS with 0.1% BSA at 4°C overnight. The membranes were washed four times with PBS before hybridizing with secondary antibody. Signals were developed by using Amersham ECL™ Western Blotting System (GE Healthcare).

### Flow cytometry

The TSH receptor cell surface expression level was quantified on a FACS flow cytometer. Transfected cells were washed twice with PBS and transferred into Falcon tubes. Cells were washed once with PBS containing 0.1% BSA and then incubated at 4°C for 1 hour with a 1∶400 dilution of a rabbit anti-human TSHR antibody (H-115). After rinse, the cells were washed twice with PBS and incubated at 4°C for 1 hour with a 1∶200 fluorescein-FITC mouse anti- rabbit IgG (2A9) (Santa Cruz Biotech). Before FACS analysis (FACscan, BD Biosciences), cells were washed twice and then fixed with 1% paraformaldehyde. The level of wild-type TSHR was set as 100% and receptor expression of the mutants was calculated according to the level of wild-type TSHR. Cell-cycle data was analyzed using the ModFIT software version 3.0 (Verity Software House, ME).

### cAMP accumulation assay

To determine cAMP, the culture medium was removed 48 hours after transfection. Thereafter, cells were incubated at 37°C for 60 min in fresh Krebs-Ringer-HEPES buffer, 0.5% BSA and the indicated concentrations of bovine (b) TSH (Sigma). The medium was removed and the reaction was stopped by adding 0.1 M HCl. Supernatants were collected, dried, resuspended in water, and then diluted appropriately for cAMP measurements. cAMP content of the cell extracts was determined using the cAMP Parameter Assay Kit (R&D Systems) according to the manufacturer's instructions.

### Inositol phosphate assay

To measure IP, transfected COS-7 cells in 6-well culture plates (2×10^5^/well) were allowed to grow for 24 hours. Thereafter, cells were washed three times with serum-free DMEM without antibiotics. After being washed with PBS, cells were incubated for 30 min in 10 mM LiCl at 37°C and then were exposed for 15 min to various concentrations of bTSH. The reactions were terminated by addition of ice-cold perchloric acid to a final concentration of 2.5%. The samples were centrifuged at 2000×g for 15 min at 4°C to remove perchloric acid precipitate. TSH-induced increases in intracellular IP levels were determined by D-myo-Inositol 1, 4, 5-trisphosphate (IP_3_) [^3^H] assay system (GE Healthcare).

### Statistical analysis

The one-way analysis of variance (ANOVA) was used for group comparisons of different treatments. Adjustment for multiple pairwise comparisons was accomplished using the adaptation of the Tukey method in conjunction with an overall 0.05 level of significance. The results were reported after adjustment for multiple comparisons. All analyses were implemented using SPSS 13.0 for Windows.
